# Acupuncture for insomnia symptoms in hypertensive patients: a systematic review and meta-analysis

**DOI:** 10.3389/fneur.2024.1329132

**Published:** 2024-02-19

**Authors:** Jieying Zhang, Xuancheng Zhou, Hailun Jiang, Weiming Zhu, Hao Chi, Lai Jiang, Shengke Zhang, Jinyan Yang, Shizhe Deng, Boxuan Li, Bifang Zhuo, Menglong Zhang, Beidi Cao, Zhihong Meng

**Affiliations:** ^1^First Teaching Hospital of Tianjin University of Traditional Chinese Medicine, Tianjin, China; ^2^Graduate School, Tianjin University of Traditional Chinese Medicine, Tianjin, China; ^3^National Clinical Research Center for Chinese Medicine Acupuncture and Moxibustion, Tianjin, China; ^4^Clinical Medical College, Southwest Medical University, Luzhou, China

**Keywords:** acupuncture, insomnia, hypertension, complementary and alternative therapies, sleep disorders

## Abstract

**Purpose:**

In the realm of pain management, traditional Chinese medicine, specifically acupuncture, has garnered increasing attention. This meta-analysis pioneers the evaluation of acupuncture’s effectiveness in treating insomnia among hypertensive patients.

**Methods:**

We conducted a comprehensive search across several databases—PubMed, Web of Science, Cochrane Library, WANFANG, China National Knowledge Infrastructure (CNKI), Sinomed, and the Chinese Journal of Science and Technology (VIP). Additionally, forward and backward articles of studies published from the inception of these databases until 10 September 2023, were reviewed. This systematic review and meta-analysis included all randomized controlled trials (RCTs) focusing on acupuncture for insomnia in hypertensive patients, without imposing language or date restrictions. We rigorously assessed all outcome measures reported in these trials. The evidence was synthesized by calculating the difference between mean differences (MD) in symptom change. The quality of the evidence was determined using the Cochrane Risk of Bias tool. This study is registered with PROSPERO under number CRD42023461760.

**Results:**

Our analysis included 16 RCTs, comprising 1,309 patients. The findings revealed that acupuncture was significantly more effective than the control group in reducing insomnia symptoms, as indicated by a greater decrease in the PSQI score (MD = −3.1, 95% CI [−3.77 to −2.62], *p* < 0.00001). Additionally, improvements in both systolic and diastolic blood pressure were more pronounced in the acupuncture group compared to the control group (SBP: MD = −10.31, 95% CI [−16.98 to −3.64], *p* = 0.002; DBP: MD = −5.71, 95% CI [−8.19 to −3.23], *p* < 0.00001). These results suggest that acupuncture not only improves sleep quality but also lowers blood pressure in patients suffering from hypertension and insomnia. Further research is warranted to elucidate optimal acupuncture points and the duration of treatment for maximized therapeutic effect.

**Systematic review registration:**https://www.crd.york.ac.uk/prospero, CRD42023461760.

## Background

Insomnia, a common sleep disorder characterized by difficulties in both falling asleep and staying asleep, accompanied by daytime dysfunction (1), affects approximately 30% of the global population, with at least one symptom of insomnia being experienced (2). This condition exerts a detrimental impact on both physical and mental health (3). Studies have demonstrated a direct link between shortened sleep duration, chronic insomnia disorders, and an increased risk of conditions such as obesity, high blood pressure, and all-cause mortality (4). Furthermore, persistent sleep problems have been associated with an increased risk of recurrent depression, and insomnia is considered a significant contributing factor to the risk of suicide (5, 6). Hypertension is recognized as a major contributor to the global burden of disease and mortality. The number of individuals with hypertension and its prevalence worldwide is expected to continue to rise over the next decade (7). Notably, symptoms of insomnia are frequently reported by patients with hypertension. Studies indicate that these patients have a relatively high risk of developing insomnia, with risk ratios (RR) ranging from 1.5 to 3.18 (8).

The conventional approach to treating patients with both hypertension and insomnia often involves the combined use of antihypertensive medications and sleep aids (9). However, due to concerns about dependence and other potential side effects, patients often show reluctance toward conventional sleeping medications, limiting their long-term clinical use. Among alternative therapies, acupuncture stands out as a popular and safe treatment option (10). Acupuncture, recognized for its effectiveness in treating a range of clinical disorders, particularly those related to neuroendocrine imbalances such as menopause, depression, and insomnia, operates by stimulating specific body points to regulate heart and brain functions (11). Acupuncture operates by stimulating specific points on the body to regulate the functions of the heart and brain.

Numerous clinical studies, notably randomized controlled trials (RCTs), have explored acupuncture’s potential as an intervention for insomnia. These studies have consistently reported positive outcomes associated with the use of acupuncture in treating this sleep disorder. Research findings suggest that acupuncture treatments are effective in reducing sleep latency, and they also contribute to an increase in sleep duration and improvement in sleep efficiency (12). This body of evidence underscores the therapeutic value of acupuncture in addressing insomnia, providing a compelling case for its inclusion in treatment plans for individuals struggling with sleep disturbances.

Despite the growing body of clinical evidence, no meta-analytic studies have yet focused on the effectiveness of acupuncture in treating insomnia specifically in hypertensive patients. This gap highlights the need for more targeted meta-analyses, especially as clinical trials evolve. Our proposed meta-analysis aims to fill this void by assessing the effectiveness of acupuncture for insomnia symptoms in hypertensive patients, using clearly defined outcome measures. By covering all clinical studies to date on this subject, our meta-analysis will provide valuable insights and inform future clinical treatment strategies. This will be particularly beneficial for physicians seeking effective methods to control insomnia in patients with hypertension.

## Methods

### Search strategy

For our systematic review and meta-analysis, we extensively searched numerous literature databases including PubMed, Web of Science, Cochrane Library, WANFANG, China National Knowledge Infrastructure (CNKI), Sinomed, and Chinese Journal of Science and Technology (VIP). Our search aimed to identify randomized controlled trials (RCTs) examining the effects of acupuncture on insomnia, spanning from the inception of each database to 10 September 2023. The search strategy was specific, utilizing terms related to “acupuncture,” “hypertension,” and “insomnia.” For interventions, we included keywords such as ‘acupuncture OR electro-acupuncture OR electrosurgical needle OR fire needle OR Fire needles’. The disease-related keywords comprised ‘Hypertension OR Blood Pressure, High OR Blood Pressures, High OR High Blood Pressure OR High Blood Pressures’ and ‘Disorders of Initiating and Maintaining Sleep OR DIMS (Disorders of Initiating and Maintaining Sleep) OR Early Awakening OR Nonorganic Insomnia OR Primary Insomnia OR Transient Insomnia OR Rebound Insomnia OR Secondary Insomnia OR Sleep Initiation Dysfunction OR Dysfunctions, Sleep Initiation OR Sleeplessness OR Insomnia Disorder OR Insomnia OR Chronic Insomnia OR Psychophysiological Insomnia’. Initially, the search utilized intervention keywords to gather relevant studies, followed by a second step employing hypertension and insomnia-related terms. The results from these two steps were then combined. All identified articles from various databases were consolidated into article management software (EndNote, version 20) for further analysis. We did not impose any specific restrictions on article types. Additionally, a thorough review of all relevant previously published meta-analyses and their reference lists was conducted. To our knowledge, there have been no recent updates on this topic, which substantiates our claim regarding the absence of recent reports in this field. The detailed search strategies employed in this study are documented in Supplementary File 1.

### Literature selection

We applied the following set of inclusion criteria during the report selection process (13, 14): (1) Patients were diagnosed with “Hypertension and Insomnia” based on explicit diagnostic (inclusion) criteria. The diagnostic criteria for hypertension are as referred to in China’s Guidelines for Prevention and Treatment of Hypertension (2018 Revision) And the following criteria are referenced in the diagnosis of insomnia: American diagnostic and statistical manual of mental disorders, fifth edition (DSM-V), classification and diagnostic criteria for Chinese mental disorders (CCDM), the diagnostic and therapeutic criteria for traditional Chinese medicine syndromes (DTCTCMS), guidelines for traditional Chinese medicine (new drug) clinical research (GTCMCR), and other commonly used diagnostic criteria. This criterion was irrespective of age, gender, duration, or source of cases, and patients did not have any other concurrent diseases. (2) These reports are randomized controlled trials that investigated the use of acupuncture (including needling, pointers, etc.) as a therapeutic intervention. Research implementation of acupuncture is not restricted in terms of manipulation and specific acupuncture points. (3) Control: this refers to any type of control group, including conventional Western medicine, routine nursing, or blank control. (4) Outcomes: at least one of the following scales was required to be included in the evaluation of sleep quality: Pittsburgh Sleep Quality Index (PSQI), the efficiency of the diagnostic and therapeutic criteria for TCM syndromes, the efficiency of guidelines for TCM (new drug) clinical research, sleep status self-assessment scale, or other inferable data mentioning insomnia and acupressure for carrying out meta-analysis; and systolic and diastolic blood pressure were used to evaluate blood pressure. Exclusion criteria encompassed (13): (1) Studies involving animal experiments, (2) Repetitive experiments, (3) Studies with incomplete data (e.g., missing sections like conference abstracts), (4) Studies published before the year 2000, as these often do not meet current standards of research quality and methodology.

### Data collection

First, two researchers independently reviewed titles and abstracts based on predetermined inclusion and exclusion criteria, and then read the full text after excluding obviously irrelevant literature. The final included literature was identified after further screening, after which the two researchers extracted data without knowledge of each other’s reviews. Finally, the results were cross-checked. These discrepancies were resolved through consensus with the third researcher.

Data extraction included the following: (1) general information: first author, year of publication, subject of literature, etc.; (2) study characteristics: baseline comparability, sample size, sex ratio, intervention, etc.; (3) outcome metrics; and (4) factors associated with assessing risk of bias.

### Quality assessment

The risk of bias in the included randomized controlled trials was assessed using the revised Cochrane Risk of Bias Tool (RoB-2). This evaluation addressed several key aspects: random sequence generation and allocation concealment (both related to selection bias), blinding of participants and personnel (performance bias), blinding of outcome assessment (detection bias), incomplete outcome data (attrition bias), selective reporting (reporting bias), and other potential biases. Each aspect was categorized based on the level of bias risk: low, unclear (indicating some concerns), or high. For instance, in assessing random sequence generation, a high risk of bias was attributed to methods prone to error, such as sorting by date of birth or outpatient number, whereas a low risk was associated with more reliable methods like random number tables, computer-generated random sequences, or coin flips (13–17). The findings from this comprehensive bias assessment were then visually represented using Revman 5.4 software, offering a clear graphical depiction of the potential biases within these trials.

### Statistical analysis

The outcomes, including the significantly efficient rate, efficacy rate, and adverse reactions, alongside the sample sizes of the investigated studies, were input into the Revman software for conducting meta-analysis. The results were then visualized through forest plots. The level of heterogeneity was evaluated using the I^2^ index, where values up to 30% indicated mild heterogeneity, 31%–50% suggested moderate heterogeneity, and values exceeding 50% indicated substantial heterogeneity. In cases where effects displayed heterogeneity (I^2^ > 50%), a random effects model was employed for the analysis. Conversely, a fixed effects model was utilized when the data appeared to be homogeneous. The calculated outcome measures and their corresponding 95% confidence intervals (CI) were illustrated in the forest plot. To determine statistical significance, a value of *p* less than 0.05 was considered indicative. Sensitivity analysis of the study using a case-by-case culling approach. Publication bias was estimated with a funnel plot (13, 14, 16, 17).

## Results

### Search results

**Figure 1:**
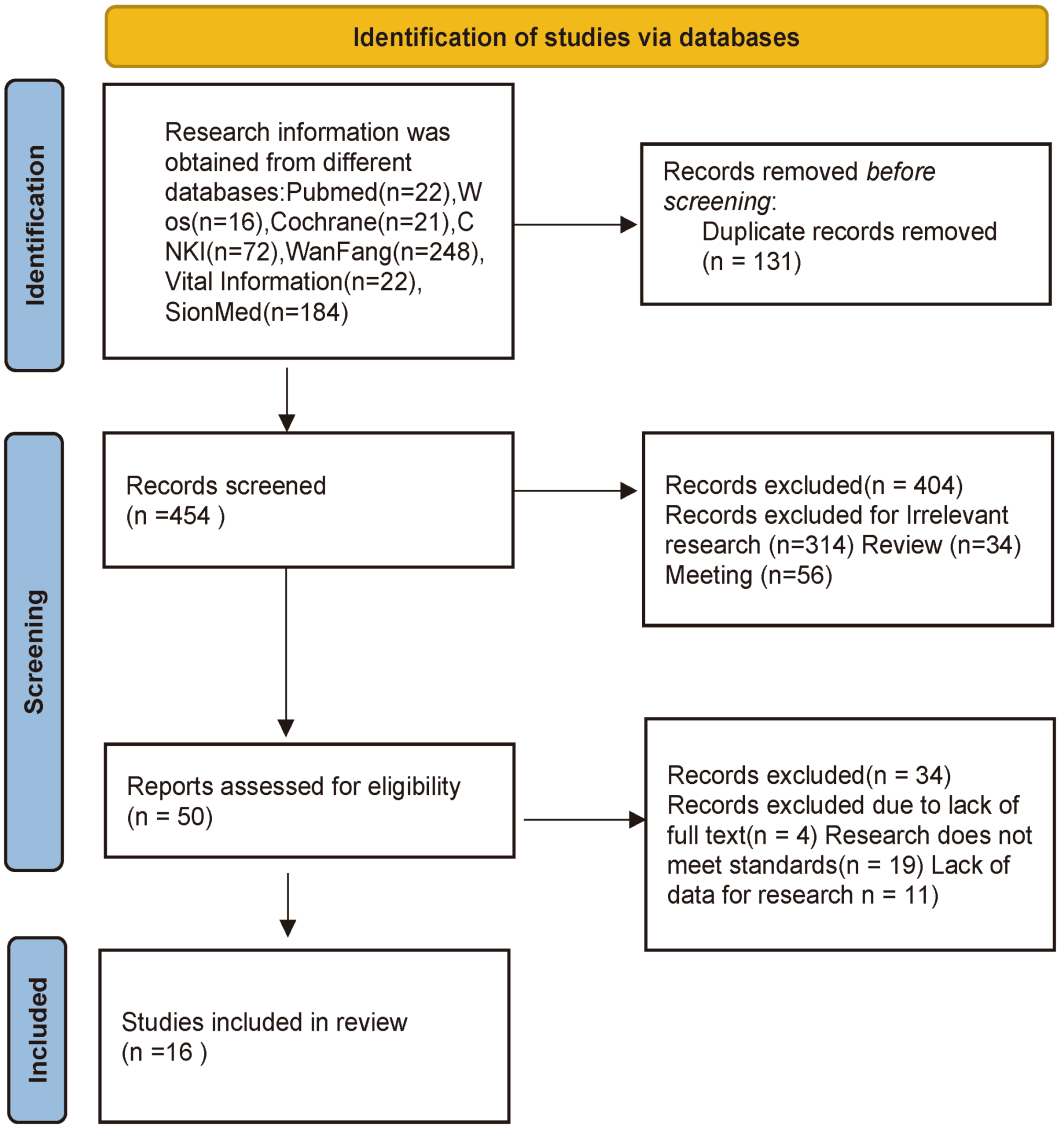
Flow diagram showing the screening and selection process of reports to be included in the meta-analysis.

At the outset, our search using the designated terms yielded a total of 585 potential research articles. Among these, 131 duplicate studies were eliminated through EndNote 20. Upon reviewing the titles and abstracts, 314 studies were identified as irrelevant and subsequently excluded. Furthermore, 90 articles were discarded due to their nature as reviews or conference materials. Subsequently, a thorough examination of the full text was conducted for 50 articles. Among these, 23 were excluded for reasons such as involving excessive time since publication, being retrospective studies, or not being pertinent to hypertension and insomnia. An additional 11 studies were excluded due to insufficient data. Ultimately, after careful scrutiny, a total of 16 clinical studies met the criteria and were deemed suitable for inclusion in the meta-analysis Figure 1.

### Characteristics of the included studies

The 16 included trials contained a total of 1,309 patients, 673 in the acupuncture group and 636 in the control group (18–33). Among the 16 acupuncture studies, one study performed direct acupressure instead of acupuncture for acupoint stimulation (21), three studies used other traditional Chinese medical treatments based on acupuncture (22, 24, 30). One study used Qiju dihuang pills in conjunction with acupuncture (22), and two other studies used acupoint compresses in conjunction with acupuncture (24, 31). In four studies the control group used other treatments based on conventional drugs. Primarily, the control group was treated with herbal medicine (19, 20, 25, 33). For the diagnosis of hypertension, the Chinese Guidelines for the Prevention and Treatment of Hypertension (18–24, 26, 30–33) were used in 12 studies. The diagnostic criteria for hypertension were as follows: systolic blood pressure ≥ 140 mmHg and/or diastolic blood pressure ≥ 90 mmHg. Hypertension was diagnosed if this criterion was met in multiple measurements. For the diagnosis of insomnia, six studies used the Chinese Guidelines for the Diagnosis and Treatment of Insomnia in Adults (19, 22, 23, 27, 31, 33), with the following diagnostic criteria: (1) Subjective description: patients subjectively felt sleep problems, including difficulty in falling asleep, difficulty in maintaining sleep, early awakening, or poor quality of sleep. (2) Duration: these sleep problems should have persisted for at least 3 months. (3) frequency: insomnia problems should occur at least 3 times a week. (4) impact on daily life: insomnia problems affect the patient’s daily life, social activities, studies or ability to work. (5) exclusion of other causes: insomnia is not caused by other medical or psychiatric disorders, e.g., depression, anxiety disorders, substance abuse, etc. Table 1 shows the main characteristics of the included studies: sample size of the treatment and control groups, age of the patients in the treatment and control groups, treatment chosen in the treatment group, treatment used in the control group, and duration of insomnia and hypertension. For the efficacy criteria of the included studies, 13 studies (18, 19, 21–30, 32) assessed SBP vs. DBP, and 12 studies (19–21, 23, 25–29, 31–33) assessed PSQI scores.7 studies (18, 19, 22–24, 31, 33) evaluated the efficacy of the treatment of insomnia using the therapeutic criteria of the TCM syndrome. Table 2 shows the included outcome metrics for the included studies.

**Table 1:** Characteristics of included studies.

Author	Total number of persons included (males/females)	Age (years)	Research design	Treatment group	Control group	Duration of hypertension (years)	Duration of insomnia (years)	Acupuncture point
Chen et al. 2020	T:40(15/25)	T:59.13 ± 8.10	RCT	Control group + acupuncture	Using conventional interventions, including antihypertensive medication, dietary guidance, and health education	10.24 ± 6.7	6.31 ± 0.7	Shenmen (HT7), Taixi (KI3)
	C:40(14/26)	C:57.65 ± 7.2						
Ding et al. 2022	T:46(15/31)	T:65 0.64 ± 2.39	RCT	Control group + acupuncture	Conventional drugs and Chinese herbal soup	T:6.48 ± 0.43 C:6.51 ± 0.47	T:14.26 ± 3.0 C:14.38 ± 3.15	Sanyinjiao (SP6), Neiguan (PC6), Shenmen (HT7), Baihui (DU20), Yintang (DU29), Shenting (DU24)
	C:44(16/28)	C:65 0.42 ± 2.45						
Guo et al. 2016	T:50(28/22)	T:70.61 ± 4.22	RCT	Control group + acupuncture	Amlodipine tablets and ozone therapy	T:8.89 ± 6.35 C:9.12 ± 6.64	NA	Fengchi (GB20)
	C:50(27/23)	C:70.99 ± 4.17						
Han 2018	T:30(17/13)	T:64.112 ± 10.54	RCT	Control group + acupoint stimulation	Benzodiazepine	T:13.12 ± 3.36 C:12.24 ± 3.41	NA	Shenmen (HT7), Shenting (DU24), Peaceful Sleep, Zhongwan (RN12), Xiawan (RN10), Zusanli (ST36)
	C:30(16/14)	C:63.25 ± 11.42						
Huanget a l. 2023	T:30(9/21)	T:70.83 ± 9.35	RCT	Control group + acupuncture and Lycium Chrysanthemum Di Huang Pills	Alprazolam tablets	NA	NA	Baihui (DU20), Four Alert Spirit Points, Neiguan (PC6), Shenmen (HT7), Sanyinjiao (SP6), Taixi (KI3)
	C:30(10/20)	C:68.23 ± 4.98						
Kong 2015	T:45(24/21)	T:55.82 ± 4.17	RCT	control group + acupuncture	Zopiclone Capsules	T:17.21 ± 6.83\u00B0C:16.90 ± 6.33	NA	Renying (ST9), Hegu (LI4), Taichong (LR3), Quchi (LI11), Zusanli (ST36), Four Alert Spirit Points
	C:45(23/22)	C:56.07 ± 5.25						
Lin et al. 2019	T:60	NA	RCT	Control group + acupuncture and acupoint stimulation	Amlodipine benzenesulfonate, eszopiclone tablets	NA	NA	Shenmen (HT7)
	C:60							
Lin et al. 2021	T:30(16/14)	T:72.98 ± 3.21	RCT	Control group + acupuncture	conventional drugs and Six-flavored Dihuang Pill	T:8.98 ± 2.90 C:9.45 ± 3.10	NA	Sanyinjiao (SP6), Shenmen (HT7), Shenting (DU24), Hegu (LI4), Neiguan (PC6), Zutonggu (BL66), Taichong (LR3)
	C:30(14/16)	C:71.99 ± 2.09						
Ma 2021	T:31(18/13)	T:47.40 ± 5.22	RCT	Control group + acupuncture	Amlodipine benzenesulfonate, eszopiclone tablets	T:3.09 ± 0.4 C:3.04 ± 0.42	NA	Shenmen (HT7), Shenting (DU24), Peaceful Sleep, Zhongwan (RN12), Xiawan (RN10)
	C:33(17/16)	C:48.53 ± 5.73						
Wang et al. 2023	T:30(18/12)	T:52.33 ± 12.91	RCT	Control group + acupuncture	Amlodipine benzenesulfonate	T:5.90 ± 2.89 C:5.70 ± 2.95	T:1.23 ± 0.58 C:1.14 ± 0.43	Baihui (DU20), Yintang (DU29), Taichong (LR3), Hegu (LI4)
	C:30(20/10)	C:49.33 ± 9.93						
Xu 2020	T:45(25/20)	T:46.93 ± 5.39	RCT	Control group + acupuncture	Nifedipine extended-release tablets, alprazolam tablets	NA	NA	Lingtai (DU10), Fengchi (GB20)
	C:45(26/19)	C:48.34 ± 5.62						
Ye et al. 2019	T:25(14/11)	T:44 ± 6.5	RCT	Control group + acupuncture	conventional drugs	T:4.81 ± 0.82 C:4.78 ± 0.84	NA	Lingtai (DU10), Fengchi (GB20)
	C:25(15/10)	C:43.8 ± 6.6						
Zhang et al. 2022	T:50(28/22)	T:55.13 ± 4.75	RCT	Control group + acupuncture	Conventional treatment and nursing interventions	T:7.14 ± 2.32 C:7.25 ± 2.21	NA	Shenmen (HT7)
	C:50(27/23)	C:55.21 ± 4.31						
Zhao 2021	T:51 (30/21)	T:59.67 ± 9.78	RCT	Control group + acupuncture and acupoint stimulation	Nifedipine, Enalapril Maleate Tablets	NA	T:7.87 ± 5.67 C:7.9 ± 5.68	Neiguan (PC6), Shenmen (HT7), Shenting (DU24), Baihui (DU20)
	C:51 (22/29)	C:58.35 ± 9.77						
Zheng et al. 2014	T:38(14/24)	T:59.84 ± 7.2	RCT	Control group + acupuncture	conventional drugs	T:9.16 ± 5.3 C:8.43 ± 4.43	NA	Shenmen (HT7)
	C:37(12/25)	C:58.95 ± 8.29						
Zhou et al. 2019	T:72(29/43)	T:64.61 ± 8.37	RCT	Control group + acupuncture	conventional drugs and Chinese herbal soup	T:7.87 ± 6.67 C:6.12 ± 4.5	NA	Neiguan (PC6), Shenmen (HT7), Shenting (DU24), Baihui (DU20)
	C:36(14/22)	C:62.73 ± 9.85						

**Table 2:** Data on outcome indicators included in the study.

Author	SBP	DBP	PSQI scores for sleep quality	PSQI scores for sleep time	PSQI scores for time to sleep	PSQI scores
Chen et al. 2020	A:126.24 ± 8.80 B:132.76 ± 9.35	A:73.34 ± 7.09 B:76.28 ± 6.54	NA	NA	NA	NA
Ding et al. 2022	A:131 0.47 ± 4.5 B:142.73 ± 5 0.39	A:83.45 ± 1 0.91 B:89.92 ± 2.1	A:1.54 ± 0.25 B:1.88 ± 0.27	A:1.64 ± 0.19 B:1.92 ± 0.25	A:1.40 ± 0.17 B:1.79 ± 0.22	NA
Guo et al. 2016	NA	NA	A:1.33 ± 0.31 B:1.94 ± 0.27	A:1.31 ± 0.24 B:2.16 ± 0.33	A:1.73 ± 0.28 B:2.03 ± 0.31	A:8.49 ± 1.34 B:12.31 ± 1.56
Han 2018	A:128.22 ± 6.84 B:135.32 ± 5.44	A:85.35 ± 6.64 B:94.11 ± 5.66	NA	NA	NA	A:5.42 ± 0.61 B:9.13 ± 1.66
Huang et al. 2023	A:138.83 ± 11.30 B:140.93 ± 11.50	A:83.27 ± 13.07 B:84.60 ± 7.20	NA	NA	NA	NA
Kong 2015	A:134.43 ± 12.37 B:142.35 ± 14.53	A:85.48 ± 8.92 B:87.85 ± 9.02	A:1.54 ± 0.63 B:2.01 ± 0.72	A:1.19 ± 0.20 B:1.86 ± 0.41	A:1.47 ± 0.23 B:1.92 ± 0.27	A:6.93 ± 1.87 B:10.33 ± 2.75
Lin et al. 2019	A:124.2 ± 7.9 B:135.4 ± 8.4	A:82.7 ± 4.6 B:88.8 ± 3. 2	NA	NA	NA	NA
Lin et al. 2021	A:125 ± 10 B:131 ± 9	A:85 ± 6 B:89 ± 8	NA	NA	NA	A:7.29 ± 3.80 B:9.80 ± 4.07
Ma 2021	A:121.10 ± 7.68 B:126.26 ± 12.15	A:74.56 ± 9.17 B:75.66 ± 8.80	A:0.8 ± 0.5 B:1.6 ± 0.6	A:0.7 ± 0.7 B:1.5 ± 0.8	A:1.4 ± 0.6 B:2.1 ± 0.5	A:6.8 ± 2.7 B:11.1 ± 2.7
Wang et al. 2023	A:131.57 ± 8.23 B:142.47 ± 9.38	A:82.30 ± 4.58 B:87.63 ± 7.67	A:1.80 ± 0.55 B:2.10 ± 0.66	A:1.50 ± 0.73 B:1.90 ± 0.76	A:1.57 ± 0.63 B:2.00 ± 0.83	A:7.77 ± 2.87 B:10.23 ± 4.17
Xu 2020	A:110.29 ± 3.08 B:141.57 ± 3.19	A:74.32 ± 2.62 B:88.49 ± 2.17	A:1.49 ± 0.08 B:1.96 ± 0.15	NA	NA	NA
Ye et al. 2019	A:135.87 ± 3.82 B:140.02 ± 4.11	A:85.86 ± 1.87 B:89.95 ± 2.13	A:1.56 ± 0.23 B:1.89 ± 0.25	A:1.65 ± 0.20 B:1.91 ± 0.23	A:1.45 ± 0.15 B:1.78 ± 0.20	NA
Zhang et al. 2022	A:119.16 ± 7.12 B:134.62 ± 7.43	A:78.41 ± 7.53 B:89.93 ± 7.68	NA	NA	NA	NA
Zhao 2021	NA	NA	A:1.56 ± 0.23 B:1.89 ± 0.25	A:0.83 ± 0.12 B:1.55 ± 0.13	A:1.03 ± 0.21 B:1.42 ± 0.21	A:6.86 ± 1.35 B:8.82 ± 2.11
Zheng et al. 2014	A:118.61 ± 6.66 B:132.73 ± 12.22	A:73.47 ± 7.17 B:76.92 ± 8.45	NA	NA	NA	A:7.37 ± 3.98 B:9.81 ± 3.76
Zhou et al. 2019	NA	NA	NA	NA	NA	A:7.13 ± 1.96 B:10.46 ± 2.77

### Quality assessment

**Figure 2:**
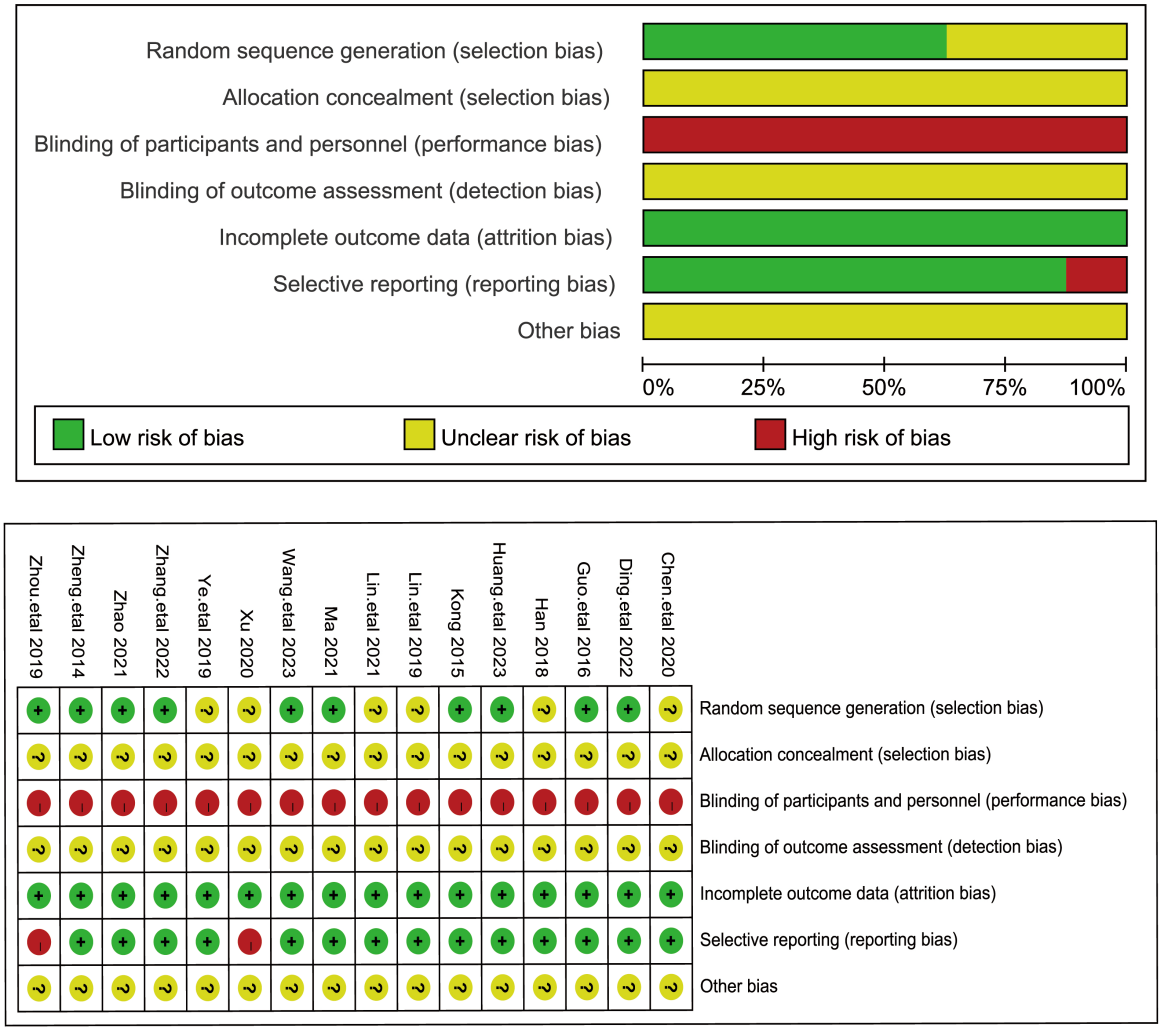
The figure represents the risk of bias assessment for the studies selected in the meta-analysis.

The results of the methodological assessment are shown in Figure 2. Ten of the 16 studies that mentioned random allocation methods were assessed as low risk due to the use of a random number table (19, 20, 22, 23, 26, 27, 30–33), and the remaining 6 studies were categorized as having an unclear risk of bias because of insufficient information provided. None of the 16 studies described the process of allocation concealment in sufficient detail and were judged to be at unclear risk of bias. Blinding of subjects or administrators could not be used in any of the 16 studies because of significant differences in the use of acupuncture treatment between treatment and control groups (18–33). The completeness of all study outcome data was judged to be at low risk of bias. Fourteen studies were categorized as having a low risk of bias for selective reporting because all prespecified endpoints were reported and were rated as having a low risk of bias for selective reporting. Two studies (18, 33) were rated at high risk of bias for selective reporting because of imperfect reporting of endpoints. In 16 studies, there were insufficient data required to judge other risks of bias (18–33).

## Results of individual studies

### Main outcome indicators

#### Systolic blood pressure

**Figure 3:**
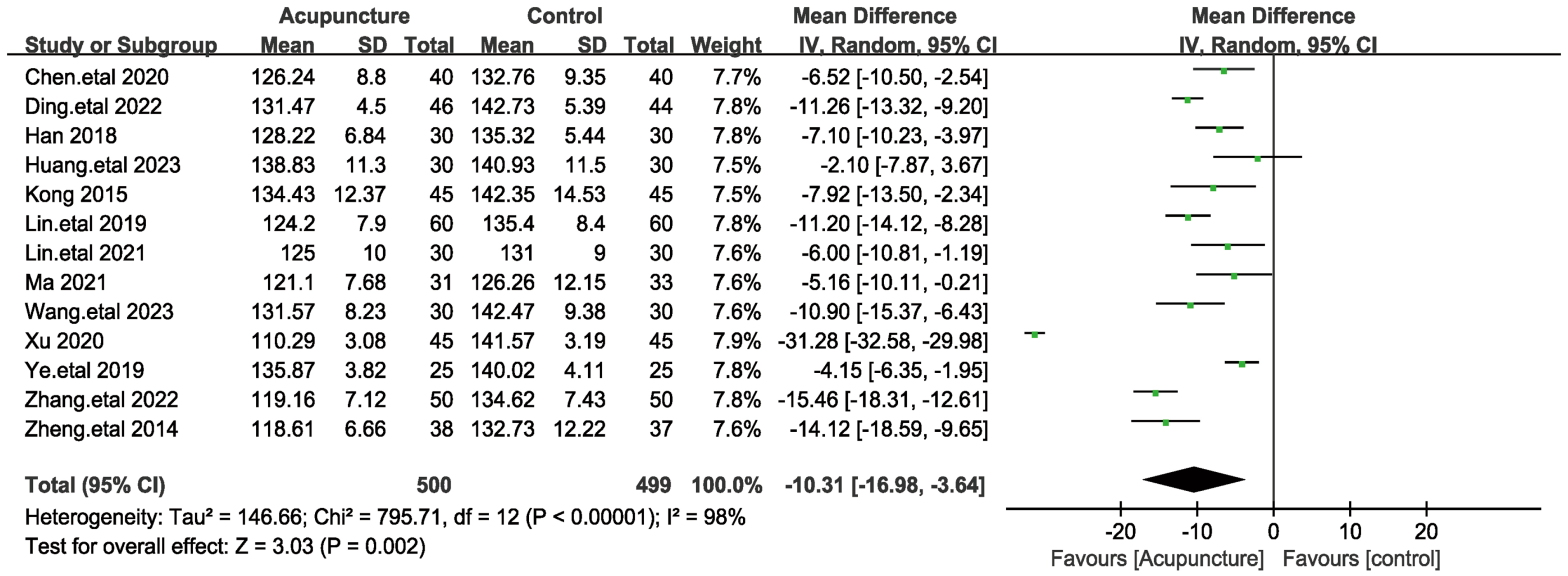
The figure represents a forest plot of the meta-analysis for systolic blood pressure (SBP). Each row represents a study and lists the name of the study, the mean systolic blood pressure and standard deviation for the acupuncture and control groups, the sample size, and the mean difference and its 95% confidence interval.

A total of 13 articles assessed systolic blood pressure, involving a total of 999 patients (18, 19, 21–30, 33). It is noteworthy that the values of Systolic blood pressure (SBP) were lower in cases where acupuncture treatment was performed. Given the large heterogeneity between these studies (I^2^ = 98%, *p* < 0.00001), a random-effects model was used. The pooled results showed (Figure 3) that the difference in SBP was statistically significant (MD = −10.31, 95% [−16.98,−3.64], *p* = 0.002). We performed a sensitivity analysis of the results using the one-by-one exclusion method, and the results were statistically significant after arbitrarily excluding one study, indicating the robustness of the results (Table 3). This result suggests that the treatment group that used acupuncture had a better effect on the improvement of blood pressure systolic compared to the control group.

**Table 3:** Sensitivity analysis of blood pressure showing pooled results after excluding one study.

Study of removal	Chen et al. 2020	Ding et al. 2022	Han 2018	Huang et al. 2023	Kong 2015	Lin et al. 2019	Lin et al. 2021	Ma 2021	Wang et al. 2023	Xu 2020	Ye et al. 2019	Zhang et al. 2022	Zheng et al. 2014
MD of SBP	−10.63 [−17.64, −3.61]	−10.22 [−17.71, −2.73]	−10.58 [−17.65, −3.51]	−10.97 [−17.89, −4.06]	−10.50 [−17.49, −3.51]	−10.23 [−17.46, −3.00]	−10.66 [−17.65, −3.68]	−10.73 [−17.70, −3.77]	−10.26 [−17.32, −3.20]	−8.68 [−11.10, −6.26]	−10.84 [−17.68, −4.00]	−9.87 [−17.18, −2.56]	−9.99 [−17.08, −2.91]
MD of DBP	−5.94 [−8.53, −3.36]	−5.58 [−8.64, −2.52]	−5.45 [−8.08, −2.83]	−6.00 [−8.56, −3.45]	−5.98 [−8.55, −3.40]	−5.65 [−8.42, −2.88]	−5.85 [−8.44, −3.25]	−6.05 [−8.61, −3.50]	−5.74 [−8.35, −3.13]	−5.17 [−6.51, −3.82]	−5.85 [−8.53, −3.17]	−5.22 [−7.82, −2.63]	−5.89 [−8.48, −3.30]

#### Diastolic blood pressure

**Figure 4:**
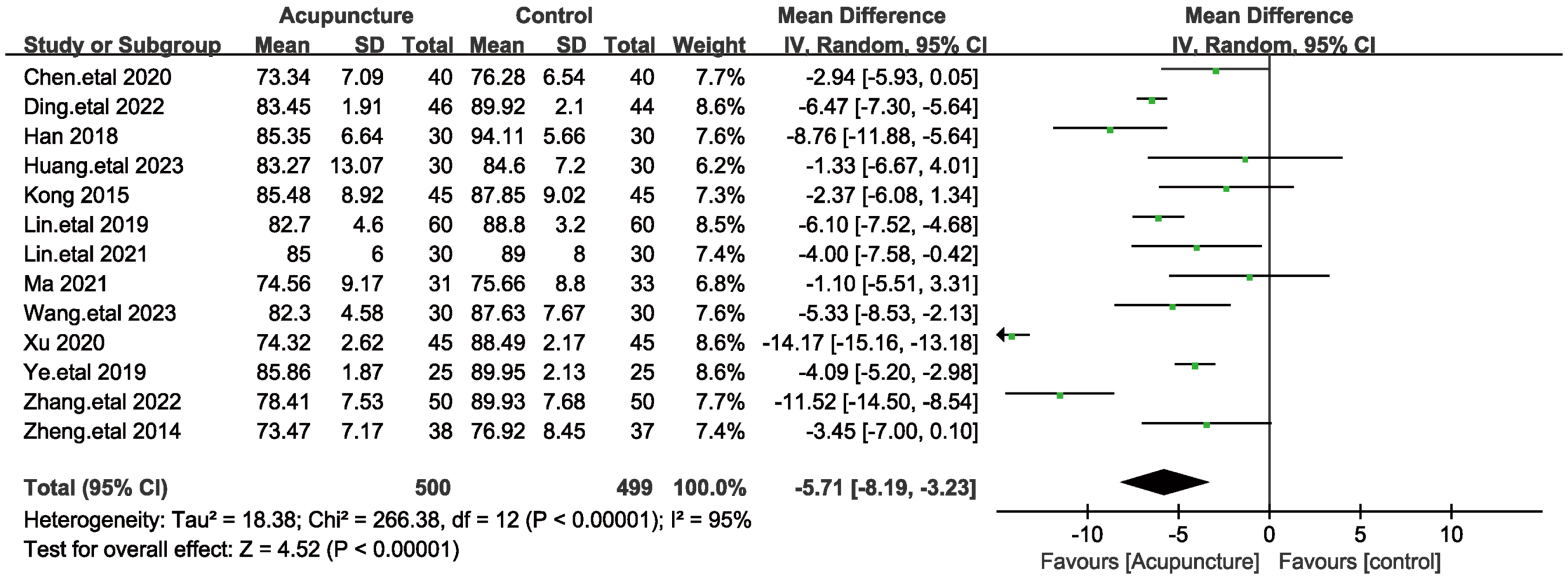
The figure represents a forest plot of meta-analysis against diastolic blood pressure (DBP).

As with systolic blood pressure, a total of 13 articles evaluated systolic blood pressure, involving a total of 999 patients (18, 19, 21–30, 32). Given the large heterogeneity among these studies (I^2^ = 95%, *p* < 0.00001), a random-effects model was used. Pooled results showed (Figure 4) that the difference in Diastolic blood pressure (DBP) was statistically significant (MD = −5.71, 95% [−8.19,−3.23], *p* < 0.00001). We performed a sensitivity analysis of the results using the one-by-one exclusion method, and the results were statistically significant after arbitrarily excluding one study, indicating the robustness of the results (Table 3). This result suggests that the treatment group that used acupuncture had a better effect on the improvement of blood pressure diastolic blood pressure compared to the control group. The results of systolic and diastolic blood pressure indicate that acupuncture treatment was effective in improving the blood pressure of the patients.

#### Total PSQI score

**Figure 5:**
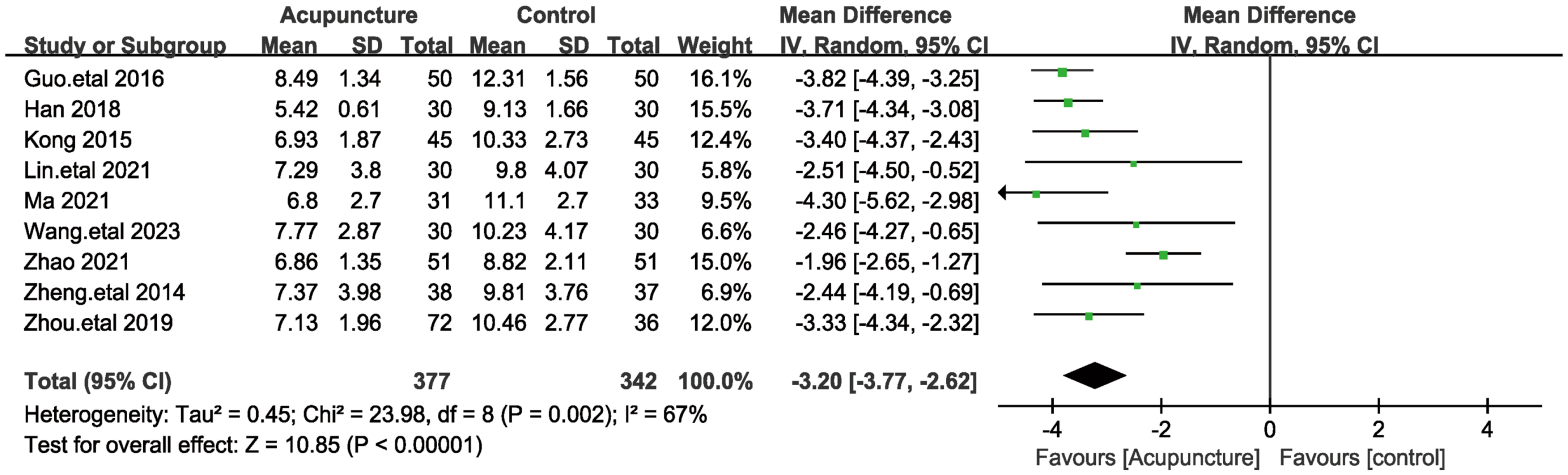
The figure represents the forest plot of the meta-analysis against total PSQI score.

**Figure 6:**
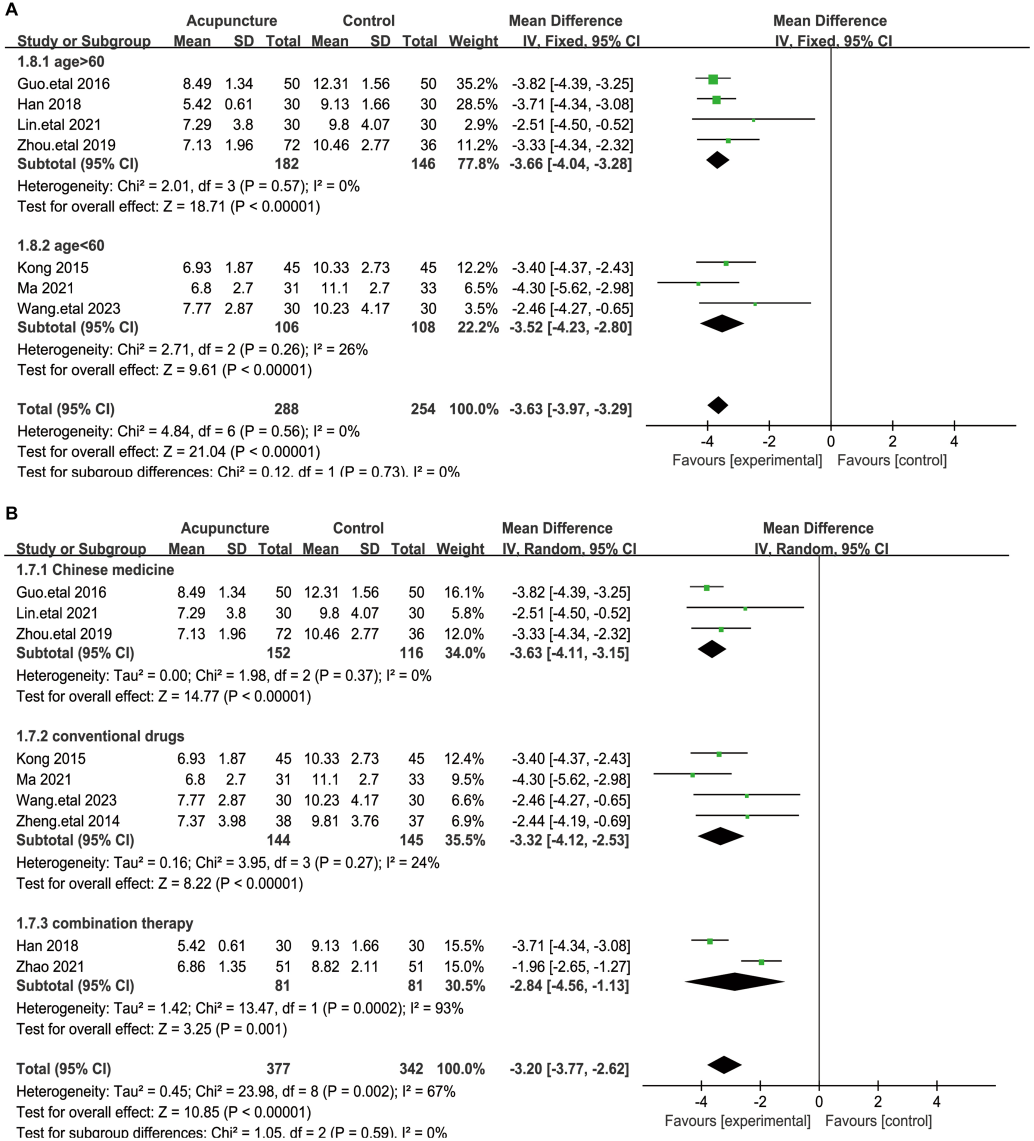
The figure represents a forest plot of subgroup analyses against total PSQI score. **(A)** Forest plots on different patient ages. **(B)** Forest plots on different interventions.

A total of 9 articles assessed systolic blood pressure, involving a total of 719 patients (20, 21, 23, 25–27, 31–33). Given the large heterogeneity among these studies (I^2^ = 67%, *p* = 0.002), a random-effects model was used. Pooled results showed (Figure 5) that the difference in total PSQI scores was statistically significant (MD = −3.1, 95% [−3.77,−2.62], *p* < 0.00001). We performed a sensitivity analysis of the results using the one-by-one exclusion method, and the results were statistically significant after arbitrarily excluding one study, indicating the robustness of the results (Table 4). This result suggests that the treatment group that used acupuncture was more effective in improving insomnia compared to the control group. In addition to this, subgroup analyses were performed based on patient age (<60 and >60 years) and method of comparison (the control group with the addition of Chinese herbs, the control group with conventional medication, and the treatment group with a combination of acupuncture and other treatments; Figure 6), which showed that none of the results were significant between subgroups. Notably, there was a great deal of heterogeneity between the two studies in the subgroup of combined treatment (I^2^ = 93%, *p* = 0.002).

**Table 4:** Sensitivity analysis of PSQI score showing pooled results after excluding one study.

Study of removal	Guo et al. 2016	Han 2018	Kong 2015	Lin et al. 2021	Ma 2021	Wang et al. 2023	Zhao 2021	Zheng et al. 2014	Zhou et al. 2019
MD	−3.08 [−3.72, −2.43]	−3.10 [−3.77, −2.42]	−3.16 [−3.82, −2.50]	−3.24 [−3.84, −2.63]	−3.08 [−3.68, −2.48]	−3.25 [−3.86, −2.64]	−3.59 [−3.92, −3.25]	−3.25 [−3.86, −2.64]	−3.17 [−3.83, −2.52]

### Secondary outcome indicators

#### PSQI sleep quality score

**Figure 7:**
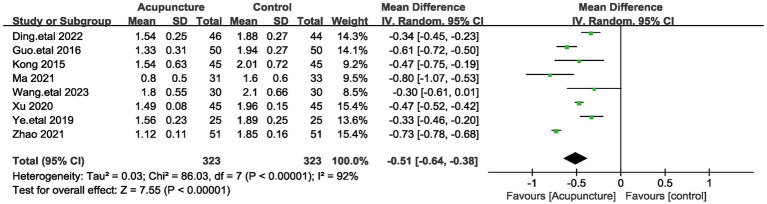
The figure represents a forest plot against the sleep quality score.

PSQI sleep quality scores were included in eight studies involving a total of 646 patients (19, 20, 23, 26–29, 31). Given the large heterogeneity among these studies (I^2^ = 92%, *p* < 0.00001), a random-effects model was used. Pooled results showed (Figure 7) that the difference in PSQI sleep quality scores was statistically significant (MD = −0.51, 95% [−0.64,−0.38], *p* < 0.00001). We performed a sensitivity analysis of the results using the one-by-one exclusion method, and the results were statistically significant after arbitrarily excluding one study, indicating that the results were robust. This result suggests that the treatment group that used acupuncture had a better effect on the improvement of sleep quality compared to the control group.

#### PSQI sleep time score

**Figure 8:**
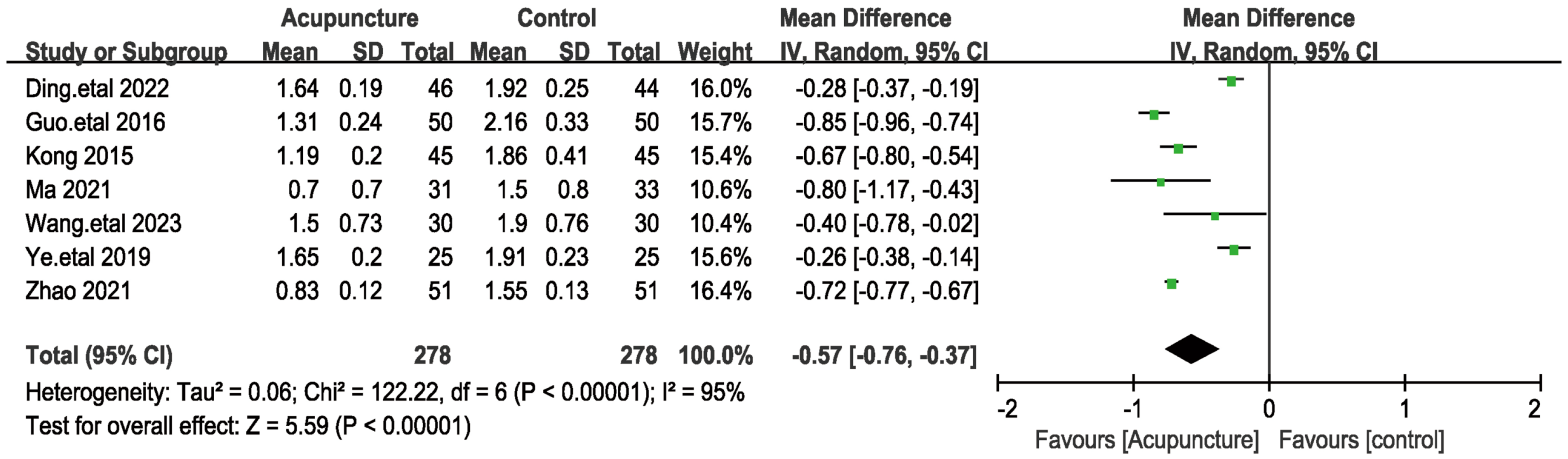
The figure represents a forest plot against the sleep duration score.

The PSQI sleep quality score was included in 7 studies involving a total of 556 patients (19, 20, 23, 26, 27, 29, 31). Given the large heterogeneity among these studies (I^2^ = 95%, *p* < 0.00001), a random-effects model was used. Pooled results showed (Figure 8) that the difference in PSQI sleep quality scores was statistically significant (MD = −0.57, 95% [−0.76,−0.37], *p* < 0.00001). We performed a sensitivity analysis of the results using the one-by-one exclusion method, and the results were statistically significant after arbitrarily excluding one study, indicating that the results were robust. This result suggests that the treatment group that used acupuncture had a better effect on the improvement of sleep duration compared to the control group.

#### PSQI time to sleep score

**Figure 9:**
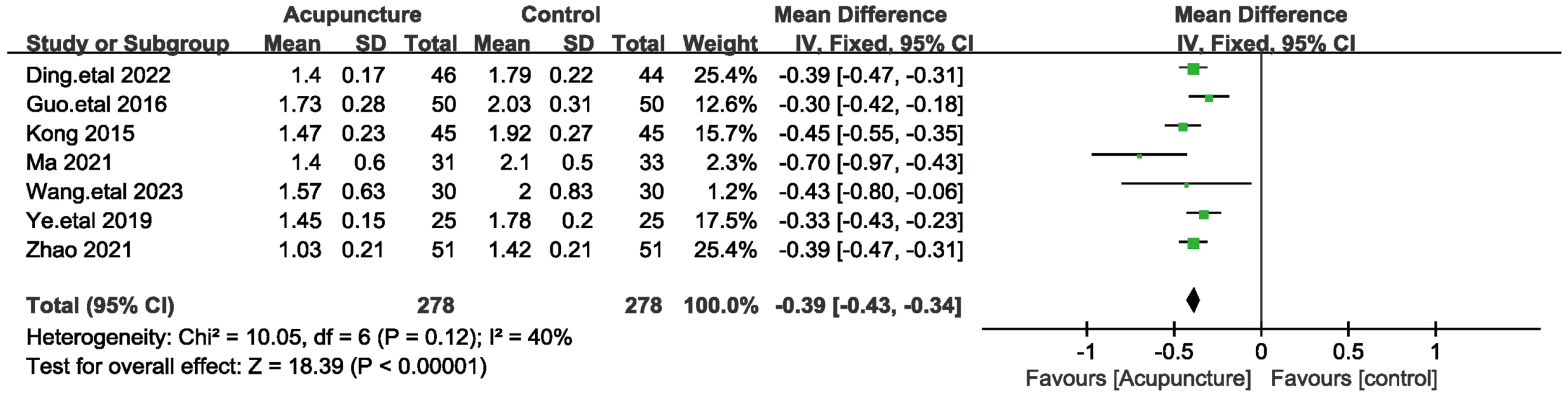
The figure represents a forest plot against the time to Sleep Score.

The PSQI time to sleep score was included in 7 studies involving a total of 556 patients (19, 20, 23, 26, 27, 29, 31). Because of the low heterogeneity among these studies (I^2^ = 40%, *p* = 0.12), a fixed-effects model was used. Pooled results showed (Figure 9) that the difference in PSQI sleep quality scores was statistically significant (MD = −0.39, 95% [−0.43,−0.34], *p* < 0.00001). We performed a sensitivity analysis of the results using the one-by-one exclusion method, and the results were statistically significant after arbitrarily excluding one study, indicating that the results were robust. This result suggests that the treatment group that utilized acupuncture had a better effect on the improvement of time to sleep compared to the control group.

#### Clinical research guidelines for Chinese medicine (new drugs) significant effectiveness and effectiveness rate

**Figure 10:**
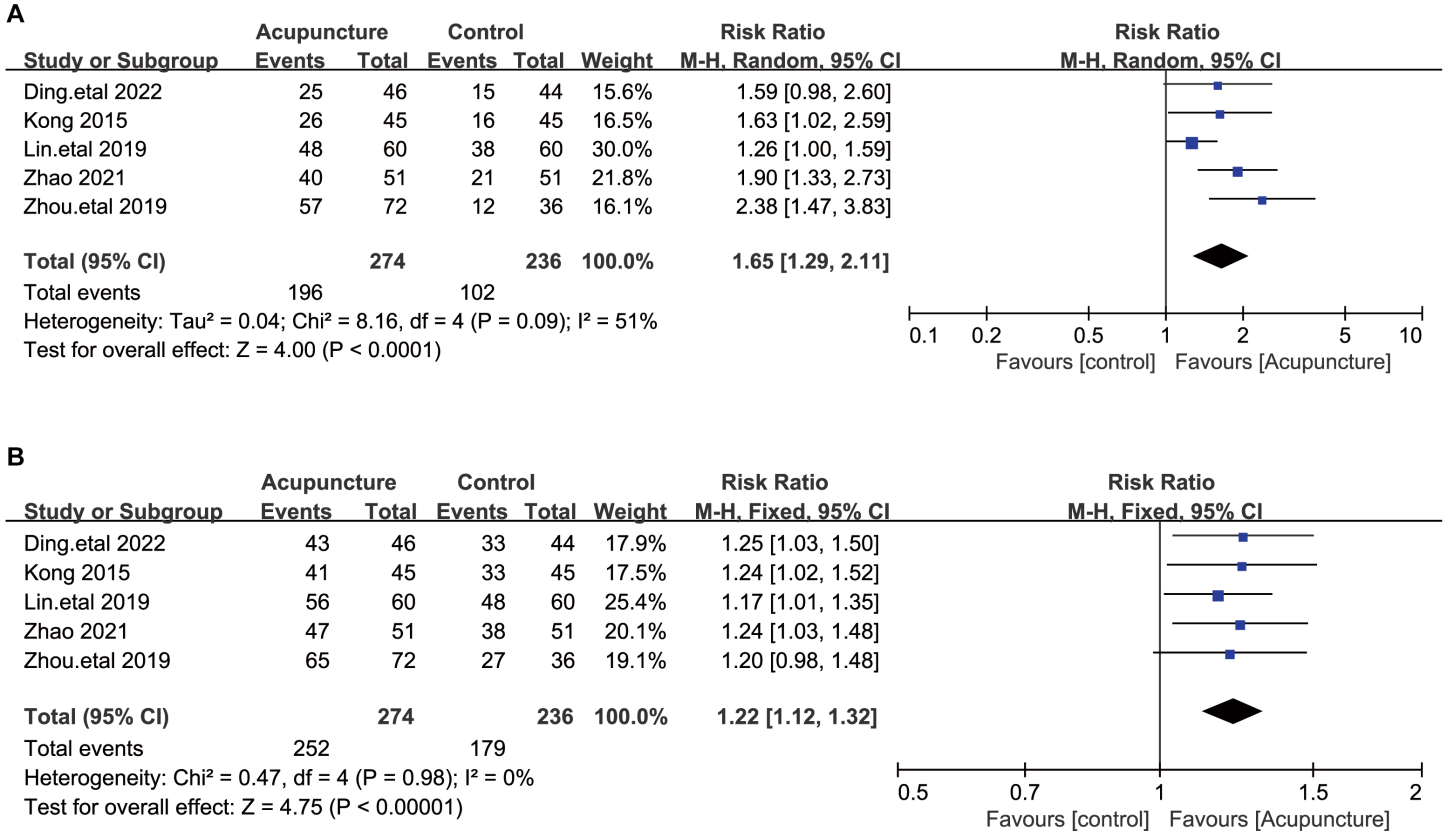
**(A)** The figure represents a forest plot against the significantly effective rates. **(B)** The figure represents a forest plot against the Overall effectiveness of insomnia treatment.

The PSQI score reduction rate was calculated by the nimodipine method in five studies using the clinical research guidelines for Chinese medicine (new drugs) (19, 23, 24, 31, 33) with reference to the relevant efficacy evaluation criteria in the Guiding Principles for the Clinical Research of New Traditional Chinese Medicines (Trial Implementation). Clinical recovery: PSOI total score reduction rate > 75%; significant effect: PSOI score reduction rate ≥ 50 and < 75%; progress: PSOI score reduction rate ≥ 25 and < 50%; ineffective: PSOI score reduction rate < 25%. We categorized cured and significantly effective together as significantly effective rates, and in view of some heterogeneity among these studies (I^2^ = 51%, *p* < 0.09), a random-effects model was used. The results showed (Figure 10A) that the combined results were statistically significant compared with the control group (RR = 1.65, 95% [1.29,2.11], *p* < 0.0001). We performed a sensitivity analysis of the results using the one-by-one exclusion method, and the results were statistically significant after arbitrarily excluding one study, indicating the robustness of the results. Next, we analyzed the total effective rate of treatment (Figure 10B), which was analyzed by meta-analysis using a fixed-effects model due to the low heterogeneity among these studies (I^2^ = 0%, *p* = 0.98). The results showed a statistically significant difference in total effective rate (RR = 1.22, 95% [1.12,1.32], *p* < 0.00001). The results of the significant effective rate and the total effective rate indicated that the treatment group that used acupuncture was more effective in the treatment of insomnia compared to the control group.

## Discussion

The treatment of insomnia in hypertensive patients typically requires a comprehensive therapeutic approach encompassing improvements in sleep habits, behavioral therapy, potential pharmacological interventions, and hypertension management. Acupuncture, a traditional Chinese medicinal treatment, is widely used in clinical practice worldwide as a traditional Chinese medicine (TCM) intervention (34–36). Previous research has provided evidence of acupuncture’s influence on the nervous system (37, 38). Investigations have also delved into the molecular mechanisms underlying acupuncture’s impact on the nervous system (39). Simultaneously, the stimulation of specific acupoints has shown therapeutic effects by modulating the expression of proteins (40). Consequently, acupuncture has progressively gained acceptance in the treatment of insomnia among hypertensive patients (41). In a previous study, acupuncture was shown to influence neuroendocrine homeostasis by modulating the vagus nerve, effectively addressing both hypertension and insomnia (42). Additionally, acupuncture has been found to enhance the levels of certain sleep-related neurotransmitters, such as serotonin and gamma-aminobutyric acid, while decreasing sleep-inhibitory neurotransmitters, like norepinephrine, in the brain (38).

Our meta-analysis results indicate that acupuncture surpasses oral western medication alone in terms of efficacy. This superiority is demonstrated through lower blood pressure profiles, reduced PSQI scores, and a higher treatment success rate within the acupuncture group, and these differences are statistically significant. To delve deeper into the comparison of acupuncture-based treatments and drug efficacy, we conducted subgroup analyses based on differences in treatment protocols between the control and acupuncture groups. Following that, we performed another subgroup analysis considering the age of patients in various studies. Notably, the results from both subgroup analyses displayed no statistically significant differences. In terms of safety, it is important to note that no serious adverse effects were reported across all studies, underscoring the excellent safety record associated with acupuncture treatment. Sensitivity analyses conducted for each outcome indicator confirmed the stability and reliability of our results.

While our findings suggest that acupuncture is a more effective treatment for insomnia in hypertensive patients compared to medication alone, it’s important to acknowledge that there was a substantial degree of heterogeneity among the studies we analyzed. This heterogeneity may arise from clinical variations, such as differences in acupoints selection and compatibility in the test group, variations in the duration of needle application, and discrepancies in the types and dosages of oral medications in the control group. The limited availability of multicenter studies suitable for inclusion in this systematic review also contributes to this limitation. Consequently, some level of bias is inherent in this analysis. To gain a more comprehensive understanding of the clinical efficacy of acupuncture in alleviating insomnia symptoms in hypertensive patients, future studies should focus on conducting prospective, multicenter, large-sample randomized controlled trials with robust study designs. We found that adverse effects were not systematically studied and documented in the included studies, which means that future studies are needed to validate the efficacy of treatment. Furthermore, these studies should aim to standardize the process of acupoint selection and treatment method in accordance with Traditional Chinese Medicine (TCM) evidence-based theories. This standardization would enhance the comparability between studies investigating such treatments and facilitate more effective quality control. Efforts are also needed to establish clinical acupuncture treatment protocols with demonstrated efficacy and high feasibility (43). This would contribute to the development of evidence-based guidelines for clinical practice.

## Conclusion

In summary, our meta-analysis results indicate that the acupuncture group exhibits greater improvements in blood pressure control, PSQI scores, and treatment efficiency when compared to the control group. This provides a theoretical basis for the use of acupuncture in the treatment of insomnia symptoms in hypertensive patients. However, due to the limitations of the available literature, there is still a need for large-sample, multicenter, and well-designed clinical trials. It may be necessary to analyze different acupoints and intervention durations to further explore the factors influencing treatment outcomes.

## Data availability statement

The original contributions presented in the study are included in the article/Supplementary material, further inquiries can be directed to the corresponding author.

## Author contributions

JZ: Conceptualization, Data curation, Formal Analysis, Methodology, Writing – original draft. XZ: Data curation, Formal Analysis, Methodology, Writing – original draft. HJ: Data curation, Formal Analysis, Writing – original draft. WZ: Conceptualization, Data curation, Writing – original draft. HC: Methodology, Visualization, Writing – original draft. LJ: Software, Writing – original draft. SZ: Methodology, Writing – original draft. JY: Visualization, Visualization. SD: Supervision, Writing – original draft. BL: Investigation, Writing – original draft. BZ: Project administration, Writing – original draft. MZ: Formal Analysis, Writing – original draft. BC: Data curation, Writing – original draft. ZM: Funding acquisition, Methodology, Resources, Supervision, Writing – original draft, Writing – review & editing.
