# Supplementary material for: Acupuncture for insomnia symptoms in hypertensive patients: a systematic review and meta-analysis
Source: Front Neurol. 2024 Feb 19;15:1329132. doi: 10.3389/fneur.2024.1329132 (PMC10910107; doi:10.3389/fneur.2024.1329132)
Supplement: Supplementary file 1 [file Data_Sheet_1.DOCX]

PubMed

(1)Hypertension OR Blood Pressure, High OR Blood Pressures, High OR High Blood Pressure OR High Blood Pressures

(2)Disorders of Initiating and Maintaining Sleep OR DIMS (Disorders of Initiating and Maintaining Sleep) OR Early Awakening OR Nonorganic Insomnia OR Primary Insomnia OR Transient Insomnia OR Rebound Insomnia OR Secondary Insomnia OR Sleep Initiation Dysfunction OR Dysfunctions, Sleep Initiation OR Sleeplessness OR Insomnia Disorder OR Insomnia OR Chronic Insomnia OR Psychophysiological Insomnia

(3) acupuncture OR Electroacupuncture OR electro-acupuncture OR electrosurgical needle OR fire needle OR Fire needles

(4)(1) and (2) and (3)

Wos

1. ((((TS=(Hypertension)) OR TS=(Blood Pressure, High)) OR TS=(Blood Pressures, High)) OR TS=(High Blood Pressure)) OR TS=(High Blood Pressures)
2. ((((((((((((((TS=(Disorders of Initiating and Maintaining Sleep)) OR TS=(DIMS (Disorders of Initiating and Maintaining Sleep))) OR TS=(Early Awakening)) OR TS=(Nonorganic Insomnia)) OR TS=(Primary Insomnia)) OR TS=(Transient Insomnia)) OR TS=(Rebound Insomnia)) OR TS=(Secondary Insomnia)) OR TS=(Sleep Initiation Dysfunction)) OR TS=(Dysfunctions, Sleep Initiation)) OR TS=(Sleeplessness)) OR TS=(Insomnia Disorder)) OR TS=(Insomnia)) OR TS=(Chronic Insomnia)) OR TS=(Psychophysiological Insomnia)
3. (((((TS=(acupuncture)) OR TS=(Electroacupuncture)) OR TS=( electro-acupuncture)) OR TS=( electrosurgical needle)) OR TS=(fire needle)) OR TS=(Fire needles)
4. #1 AND #2 AND #3

**Cochrane**

1. ‘Hypertension’ OR ‘Blood Pressure, High’ OR ‘Blood Pressures, High’ OR ‘High Blood Pressure’ OR ‘High Blood Pressures’
2. ‘Disorders of Initiating and Maintaining Sleep’ OR ‘DIMS (Disorders of Initiating and Maintaining Sleep)’ OR ‘Early Awakening’ OR ‘Nonorganic Insomnia’ OR ‘Primary Insomnia’ OR ‘Transient Insomnia’ OR ‘Rebound Insomnia’ OR ‘Secondary Insomnia’ OR ‘Sleep Initiation Dysfunction’ OR ‘Dysfunctions, Sleep Initiation’ OR ‘Sleeplessness’ OR ‘Insomnia Disorder’ OR ‘Insomnia’ OR ‘Chronic Insomnia’ OR ‘Psychophysiological Insomnia’
3. ‘acupuncture’ OR ‘Electroacupuncture’ OR ‘electro-acupuncture’ OR ‘electrosurgical needle’ OR ‘fire needle’ OR ‘Fire needles’

万方

主题：（（“针灸”or “针刺”or “火针”or “电针”）and（“高血压”or “原发性高血压”or “继发性高血压”or “高收缩压”or “高舒张压”or “血压”）and（“失眠”or “睡眠障碍”or “入睡困难”or “不寐”or “夜间觉醒”or “早醒”））

维普

1. 针灸+针刺+火针+电针

2. 高血压+原发性高血压+继发性高血压+高收缩压+高舒张压+血压

3. 失眠+睡眠障碍+入睡困难+不寐+夜间觉醒+早醒

4.#1+#2+#3

SionMed

（1）"针灸"[常用字段:智能] OR "针刺"[常用字段:智能] OR "火针"[常用字段:智能] OR "电针"[常用字段:智能]

（2）"高血压"[常用字段:智能] OR "原发性高血压"[常用字段:智能] OR "继发性高血压"[常用字段:智能] OR "高收缩压"[常用字段:智能] OR "高舒张压"[常用字段:智能] OR "血压"[常用字段:智能]

（3）"失眠"[常用字段:智能] OR "睡眠障碍"[常用字段:智能] OR "入睡困难"[常用字段:智能] OR "不寐"[常用字段:智能] OR "夜间觉醒"[常用字段:智能] OR "早醒"[常用字段:智能]

（4）("失眠"[常用字段:智能] OR "睡眠障碍"[常用字段:智能] OR "入睡困难"[常用字段:智能] OR "不寐"[常用字段:智能] OR "夜间觉醒"[常用字段:智能] OR "早醒"[常用字段:智能]) AND ("高血压"[常用字段:智能] OR "原发性高血压"[常用字段:智能] OR "继发性高血压"[常用字段:智能] OR "高收缩压"[常用字段:智能] OR "高舒张压"[常用字段:智能] OR "血压"[常用字段:智能]) AND ("针灸"[常用字段:智能] OR "针刺"[常用字段:智能] OR "火针"[常用字段:智能] OR "电针"[常用字段:智能])

CNKI

(针灸 + 针刺 + 火针 + 电针) * (高血压 + 原发性高血压 + 继发性高血压 + 高收缩压 + 高舒张压 + 血压) * (失眠 + 睡眠障碍 + 入睡困难 + 不寐 + 夜间觉醒 + 早醒)
